# Comparing the mitochondrial genomes of *Wolbachia*-dependent and independent filarial nematode species

**DOI:** 10.1186/1471-2164-13-145

**Published:** 2012-04-24

**Authors:** Samantha N McNulty, Andrew S Mullin, Jefferson A Vaughan, Vasyl V Tkach, Gary J Weil, Peter U Fischer

**Affiliations:** 1 Infectious Diseases Division, Department of Internal Medicine, Washington University School of Medicine, Campus Box 8051, 660 S. Euclid Avenue, St. Louis, MO, 63110, USA; 2 Department of Biology, University of North Dakota, 10 Cornell St, Grand Forks, ND, 58202, USA

## Abstract

**Background:**

Many species of filarial nematodes depend on *Wolbachia* endobacteria to carry out their life cycle. Other species are naturally *Wolbachia*-free. The biological mechanisms underpinning *Wolbachia*-dependence and independence in filarial nematodes are not known. Previous studies have indicated that *Wolbachia* have an impact on mitochondrial gene expression, which may suggest a role in energy metabolism. If *Wolbachia* can supplement host energy metabolism, reduced mitochondrial function in infected filarial species may account for *Wolbachia*-dependence. *Wolbachia* also have a strong influence on mitochondrial evolution due to vertical co-transmission. This could drive alterations in mitochondrial genome sequence in infected species. Comparisons between the mitochondrial genome sequences of *Wolbachia*-dependent and independent filarial worms may reveal differences indicative of altered mitochondrial function.

**Results:**

The mitochondrial genomes of 5 species of filarial nematodes, *Acanthocheilonema viteae, Chandlerella quiscali*, *Loa loa*, *Onchocerca flexuosa*, and *Wuchereria bancrofti*, were sequenced, annotated and compared with available mitochondrial genome sequences from *Brugia malayi, Dirofilaria immitis, Onchocerca volvulus* and *Setaria digitata*. *B. malayi*, *D. immitis, O. volvulus* and *W. bancrofti* are *Wolbachia*-dependent while *A. viteae, C. quiscali*, *L. loa, O. flexuosa* and *S. digitata* are *Wolbachia*-free. The 9 mitochondrial genomes were similar in size and AT content and encoded the same 12 protein-coding genes, 22 tRNAs and 2 rRNAs. Synteny was perfectly preserved in all species except *C. quiscali,* which had a different order for 5 tRNA genes. Protein-coding genes were expressed at the RNA level in all examined species. In phylogenetic trees based on mitochondrial protein-coding sequences, species did not cluster according to *Wolbachia* dependence.

**Conclusions:**

Thus far, no discernable differences were detected between the mitochondrial genome sequences of *Wolbachia*-dependent and independent species. Additional research will be needed to determine whether mitochondria from *Wolbachia*-dependent filarial species show reduced function in comparison to the mitochondria of *Wolbachia*-independent species despite their sequence-level similarities.

## Background

Filarial nematodes are arthropod borne parasitic worms that infect hundreds of millions of people throughout the tropics and sub-tropics and are responsible for a great deal of morbidity in humans and domestic animals. Many filarial pathogens, such as the agents of lymphatic filariasis and river blindness, require a bacterial endosymbiont, *Wolbachia pipientis*, to carry out their life cycle [1-4]. In these species, depletion of the endosymbiont causes defects in growth, molting and fertility, leading to the death of the worm [5-7]. Other filarial species, some of which are very closely related to *Wolbachia*-dependent sister taxa, are naturally *Wolbachia*-free [1,2,8-10]. Thus far, there are no discernable patterns in *Wolbachia* distribution (e.g., based on host species, vector species, tissue tropism, geographic distribution, etc.), and the reasons for this disparity are poorly understood. Presumably, some genetic function(s) must be missing or reduced in *Wolbachia*-dependent worms in comparison to their *Wolbachia*-free counterparts, forcing them to rely on the bacteria as an alternative source of vital gene products. The processes underpinning *Wolbachia*-dependence are of biological and medical interest, as the *Wolbachia* products required by the dependent worm may represent useful targets for novel anti-filarial chemotherapies.

*Wolbachia* endobacteria and the eukaryotic mitochondria share many common features, including the intracellular lifestyle, obligatory mutualism, reduced genome size, vertical transmission, etc. These shared features, as well as their shared ancestry in the order Rickettsiales [11-13], lead us to hypothesize that *Wolbachia* may contribute to energy metabolism in the filarial host. Previous studies have shown that antibiotic-mediated *Wolbachia* depletion leads to upregulation in genes related to energy metabolism, including mitochondrially encoded subunits of the respiratory chain [14]. This impact on host mitochondrial gene expression, and presumably energy production, suggests that *Wolbachia* may serve as an alternative energy source or mitochondrial “supplement,” necessitating increased activity when the endosymbiont is removed. If so, differences in mitochondrial function may account for discrepancies in *Wolbachia* status in the filarial lineage.

The mitochondrial genome (mtDNA) is particularly sensitive to evolutionary pressure exerted by the *Wolbachia* infection. Vertically-transmitted *Wolbachia* are able to expand rapidly through insect populations due to the mechanisms of reproductive parasitism [15]. *Wolbachia* and mitochondria are co-transmitted. Thus, the mtDNA(s) of the first infected individual(s) presumably expand concurrently with the *Wolbachia* infection. Such *Wolbachia*-mitochondria “sweeps,” characterized by unusually low degrees of variation in the mtDNA of infected populations, have been noted in many insect species [16-20]. A similar lack of mtDNA diversity is seen in populations of *Dirofilaria immitis* (canine heartworm) in comparison to *Wolbachia*-free, non-filarial nematodes [21]. A *Wolbachia*-induced genetic bottleneck may have led to the fixation of different mtDNA types among infected filarial species as compared to uninfected species.

The mtDNA sequences of 4 species of filarial nematodes, *Onchocerca volvulus*[22]*, D. immitis*[23]*, Brugia malayi*[24]*,* and *Setaria digitata*[25], have been published. This report details the sequencing and analysis of the mtDNA sequences of 5 more species: *Acanthocheilonema viteaeChandlerella quiscali, Loa loa, Onchocerca flexuosa* and *Wuchereria bancrofti*. Studies of the distribution of *Wolbachia* within filarial nematodes have shown that the infection is prevalent among 2 of the 8 filarial subfamiles, the Onchocercinae and the Dirofilariinae [1,2]. Agreement between the phylogenies of *Wolbachia* and their filarial hosts suggests that *Wolbachia* entered the filarial lineage prior to the diversification of these 2 subfamilies [1,26]. 7 of the 9 species included in this study are members of the the Onchocercinae and Dirofilariinae. Four of these, *B. malayiD. immitisO. volvulus* and *W. bancrofti*, are *Wolbachia*-dependent [1,3,4,27]. The other 3, *A. viteaeL. loa* and *O. flexuosa*, are *Wolbachia*-free [1,8,10,28], presumably due to secondary loss of the endosymbiont [1,29]. Conversely, *C. quiscali* and *S. digitata* are *Wolbachia*-free and belong to subfamilies (Splendidofilariinae and Setariinae, respectively) that have not been shown to contain *Wolbachia*-infected species, suggesting that these subfamilies split from the lineage prior to the introduction of *Wolbachia* endobacteria [2,9].

In light of the presumed impact of *Wolbachia* on the host mitochondria, we hypothesized that the mtDNAs of *Wolbachia*-dependent filaria may differ in gene content, arrangement or sequence as compared to those found in *Wolbachia*-free species whose ancestor(s) may not have undergone a *Wolbachia*-induced genetic bottleneck or evolved in the presence of an endobacterial partner capable of affecting host energy metabolism. The purpose of the reported study was to compare mtDNA from *Wolbachia*-dependent and independent filarial species in search of sequence level differences indicative of altered mitochondrial function. Our analyses revealed no differences that could be attributed to *Wolbachia* status. Future studies will be required to discover subtler affects of *Wolbachia* on the sequence or function of filarial nematode mitochondria.

## Results

### Gene content and organization

The mtDNAs of 5 species of filarial nematodes were sequenced, annotated and deposited in Genbank (see Table 1 for accession numbers). Genome length, AT-richness and base composition of the 9 mtDNAs are compared in Table 1. The newly sequenced mtDNAs are similar in size and AT content to those of other filarial species. So far, filarial mtDNAs range in size from 13,474 bp in *O. volvulus* to 13,839 in *S. digitata* and range in AT content from 73.7% in *O. volvulus* to 77.7% in *C. quiscali*[22,25]*.*

**Table 1:** Comparison of filarial nematode mtDNAs

	*Wolbachia*-dependent species				*Wolbachia*-independent species				
Species	*B. malayi*	*D. immitis*	*O. vovlulus*	*W. bancrofti*	*A. viteae*	*L. loa*	*O. flexuosa*	*C. quiscali*	*S. digitata*
Subfamily	Onchocercinae	Dirofilariinae	Onchocercinae	Onchocercinae	Onchocercinae	Dirofilariinae	Onchocercinae	Splendidofilariinae	Setariinae
Accession Number	NC_004298	NC_005305	NC_001861	HQ184469	HQ186249	HQ186250	HQ214004	HM773029	NC_014282
Length (bp)	13,657	13,814	13,474	13,636	13,724	13,590	13,672	13,757	13,839
Length of AT-rich region (bp)	283	362	312	256	421	288	284	308	506
A%	21.60%	19.30%	19.30%	20.50%	19.60%	20.80%	20.30%	23.00%	19.40%
T%	53.90%	54.90%	54.00%	54.10%	54.00%	54.80%	53.90%	54.70%	55.70%
G%	16.80%	19.30%	19.80%	18.00%	19.30%	17.70%	18.60%	15.90%	18.20%
C%	7.70%	6.50%	6.90%	7.40%	7.20%	6.70%	7.20%	6.40%	6.70%
AT%	75.50%	74.20%	73.30%	74.60%	73.50%	75.60%	74.20%	77.70%	75.10%

Information was taken from previous studies for *B. malayi*[24], *D. immitis*[23], *O. volvulus*[22], and *S. digitata*[25]. Information regarding the *Wolbachia* status of species from various subfamilies is reported in [2].

**Figure 1:**
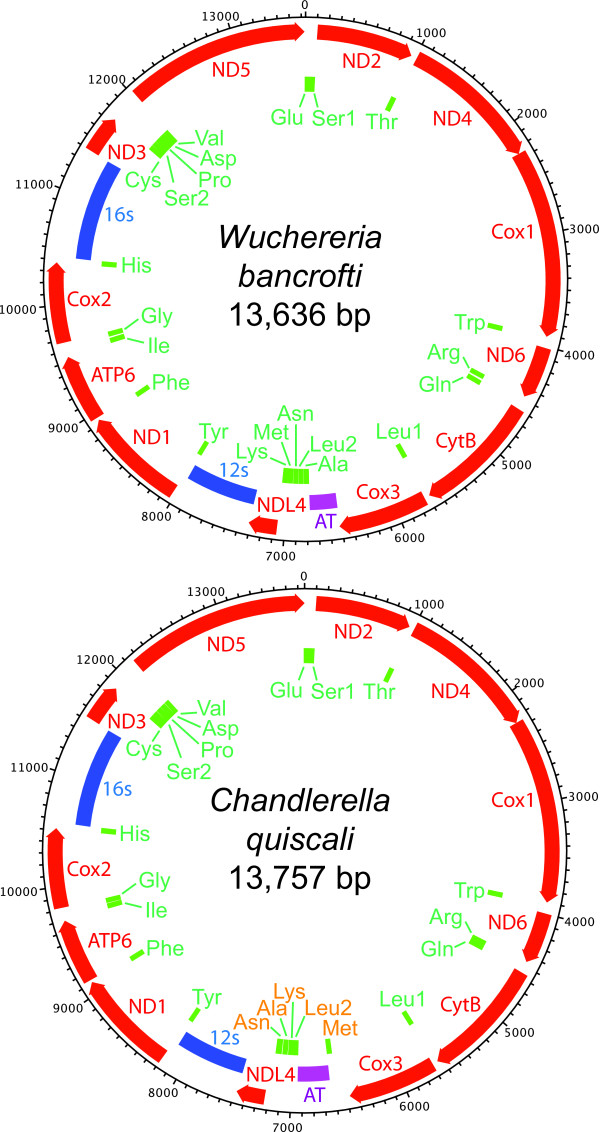
**Comparative diagrams of the mitochondrial genomes of*W. bancrofti*and*C. quiscali*.** Protein-coding genes are shown in red with arrowheads indicating directionality. rRNA and tRNA genes are shown in blue and green, respectively, and the AT-rich region is shown in purple. The diagram of the *W. bancrofti* mitochondrial genome is representative of most filarial mitochondria, as synteny is preserved in all species except *C. quiscali*. The 5 tRNA genes rearranged in *C. quiscali* are highlighted in orange.

All 9 filarial mtDNAs encode the same 12 proteins, 22 tRNAs and 2 rRNAs with very short intergenic sequences (Figure 1). These genes are encoded in the same direction, a characteristic shared by most nematode mtDNAs. Synteny is perfectly preserved in all examined species with the exception of *C. quiscali* (Figure 1). In 8 of the 9 species, 5 tRNA genes (tRNA^Ala^, tRNA^Leu2^, tRNA^Asn^, tRNA^Met^ and tRNA^Lys^) reside between the AT-rich region and NDL4. In *C. quiscali*, the tRNA^Met^ gene is positioned between Cox3 and the AT-rich region apart from the main tRNA cluster, and the order of the other 4 tRNA genes is rearranged relative to other species. For a comparisons between the mtDNA arrangement among filarial and other nematodes, see [25].

### Protein-coding genes

**Figure 2:**
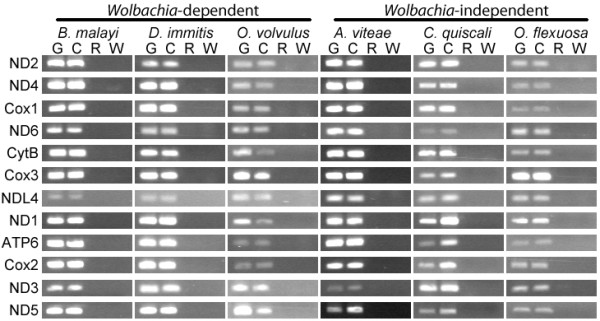
**Expression of mitochondrial protein-coding genes in six filarial nematode species.** Expression of mitochondrial protein-coding genes was assessed by reverse transcription PCR in *B. malayi*, *D. immitis, O. volvulus*, *A. viteae, C. quiscali* and *O. flexuosa*. The following templates were used for each reaction: genomic DNA (G), cDNA (C), total RNA (R) and water (W).

Twelve protein-coding genes were identified in each of the examined mtDNAs. None of these contain premature stop codons or frameshift mutations. Reverse transcription PCR reactions indicate that the predicted protein-coding genes were expressed at the RNA level in all examined species (Figure 2).

Filarial mtDNAs are extremely thymine (T)-rich (Table 1); therefore, it is not surprising that filarial mitochondria show a bias towards T-rich codons ( Additional file 1: Table S1). The most frequently used codon in all species is TTT, which encodes phenylalanine and serves as an alternative start codon in certain instances ( Additional file 1: Table S1, Table 2). The start and stop codons used by each species are listed in Table 2. Novel start codons include TGT for ND6 in *W. bancrofti*, TCT for CytB in *A. viteae*, and CCT for ND3 in *O. flexuosa*. Termination codons include TAG, TAA, and the incomplete stop codon T, which is converted to TAA upon addition 3’ poly(A) tail.

**Table 2:** Start and stop codons used in mitochondrial protein-coding genes

	*Wolbachia*-dependent species				*Wolbachia*-independent species				
	*B. malayi*	*D. immitis*	*O. volvulus*	*W. bancrofti*	*A. viteae*	*L. loa*	*O. flexuosa*	*C. quiscali*	*S. digitata*
ND2	TTA/T	ATT/TAG	ATT/TAG	TTA/T	TTT/TAG	ATT/TAA	ATT/TAG	ATT/TAG	TTT/TAG
ND4	TTG/TAA	TTG/TAG	TTG/TAA	TTG/TAA	ATG/TAA	TTG/TAG	TTG/TAG	TTG/TAA	ATG/TAA
COX1	ATT/TAG	ATT/TAG	ATT/TAG	ATT/TAA	GTT/T	GTT/T	ATT/TAA	TTG/TAA	ATT/TAG
ND6	TAT/TAA	TAT/TAG	ATT/TAG	TGT/TAA	TAT/TAG	TAT/TAG	ATT/TAA	TTG/TAG	TTG/TAA
CYTB	ATT/T	GTT/T	ATT/TAA	ATT/T	TCT/T	ATT/T	ATT/TAA	ATT/T	GTT/T
COX3	ATT/TAA	ATT/TAA	ATT/TAA	ATT/TAA	ATT/TAA	ATT/TAA	ATT/TAG	ATT/TAA	ATA/T
NDL4	GTA/TAA	GTA/TAA	TTG/TAA	GTA/TAA	GTA/T	GTA/T	TTA/T	GTT/TAA	TTG/T
ND1	TTG/T	TTG/T	TTG/T	TTG/TAA	TTG/T	TTG/T	TTG/T	TTG/T	TTG/TAA
ATP6	ATT/TAG	TTG/TAA	ATT/TAG	ATT/TAG	ATT/TAA	ATT/TAA	ATT/TAA	ATT/TAG	TTT/TAG
COX2	ATT/TAA	ATT/T	ATT/TA	ATT/TAA	ATT/TAA	ATT/TAA	ATT/TAA	ATT/TAA	ATT/TAG
ND3	CTT/TAG	CTT/T	CTT/TAG	CTT/T	CTT/T	CTT/T	CCT/T	CTT/TAG	TTT/T
ND5	TTT/TAG	TTG/TAG	TTG/TAG	TTT/TAG	TTT/TAG	TTT/TAG	TTA/TAA	TTT/T	TTT/TAG

Information was taken from previous studies for *B. malayi*[24], *D. immitis*[23], *O. volvulus*[22], and *S. digitata*[25]. Information regarding the *Wolbachia* status of species from various subfamilies is reported in [2].

### Ribosomal and transfer RNA genes

All species encode 2 rRNA genes. In all species examined, the 12s rRNA gene is positioned between NDL4 and ND1 while the 16s rRNA gene is positioned between Cox2 and ND3 (Figure 1). The exact boundaries of these genes have yet to be mapped in any filarial species.

**Figure 3:**
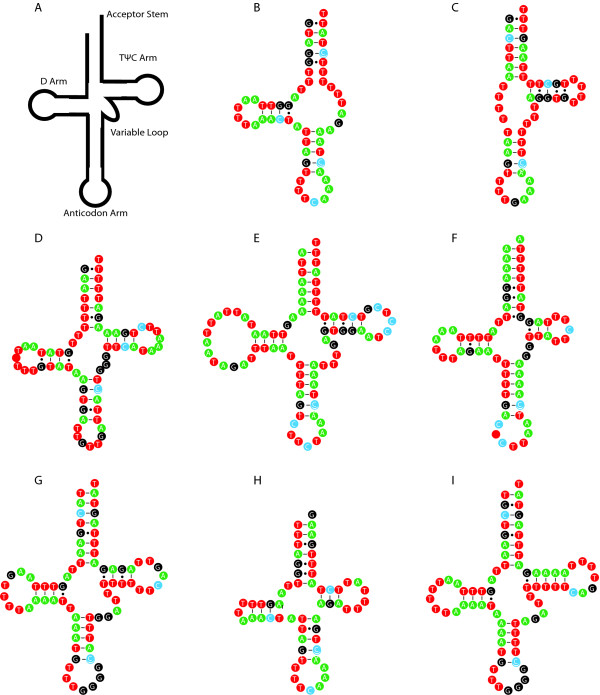
**Mitochondrial tRNA structures.** The features of a typical tRNA include the acceptor stem, D arm, TΨC arm, variable loop and anticodon arm (A). In most species, tRNA^Ser1^ and tRNA^Ser2^ contain a DHU replacement loop and TΨC arm, as in tRNA^Ser2^ from *A. viteae* (B), while all other tRNAs contain a D arm and TV replacement loop, as in tRNA^Trp^ of *A. viteae* (C). Exceptions may include tRNA^Asn^ (D) and tRNA^Ser1^ (E) from *C. quiscali*, tRNA^Lys^ (F) and tRNA^Pro^ (G) from *L. loa*, tRNA^Trp^ (H) from *O. flexuosa*, and tRNA^Pro^ (I) from *W. bancrofti*, as these structures are predicted to include both the D and TΨC arms.

All species also contain the same 22 tRNA genes. In the previously sequenced species, 20 of the 22 mitochondrial tRNAs share a common secondary structure in which the TΨC arm and variable loop are exchanged for a TV-replacement loop [22,23] (Figure 3b). Conversely, the two tRNA^Ser^ genes contain a DHU replacement loop in exchange for the typical D arm (Figure 3c) [22,23]. The predicted mitochondrial tRNA structures of *A. viteae* followed this trend exactly, as did most of the tRNAs from the other examined species. However, our predictions indicate that tRNA^Ser1^ and tRNA^Asn^ in *C. quiscali*, tRNA^Lys^ and tRNA^Pro^ in *L. loa*, tRNA^Trp^ in *O. flexuosa* and tRNA^Pro^ in *W. bancrofti* may contain both the TΨC and D loops (Figure 3d-i). The same anticodons are used in all species with two exceptions. tRNA^Pro^ uses the anticodon AGG in *O. volvulusD. immitisS. digitata* and *O. flexuosa*, while the anticodon TGG is used in other species, and tRNA^Leu1^ uses the anticodon TAA in *A. viteae* while the anticodon TAG is used in other species.

### AT rich region

The control, or AT rich, region represents the largest non-coding region in filarial mtDNAs, which are otherwise densely packed with tightly spaced or slightly overlapping protein-coding, tRNA and rRNA genes. The AT rich regions of the 9 sequenced filarial mtDNAs range in size from 256 bp in *W. bancrofti* to 506 bp in *S. digitata* (Table 1). In most species, this region is located between Cox3 and tRNA^Ala^. The unusual arrangement of tRNA genes in *C. quiscali* places its proposed 308 bp AT rich region between the tRNA^Met^ and tRNA^Leu2^ genes, leaving an additional 109 bp non-coding region between the Cox3 and tRNA^Met^ genes. The function of this secondary non-coding region is unknown.

### Phylogenetic analysis

**Figure 4:**
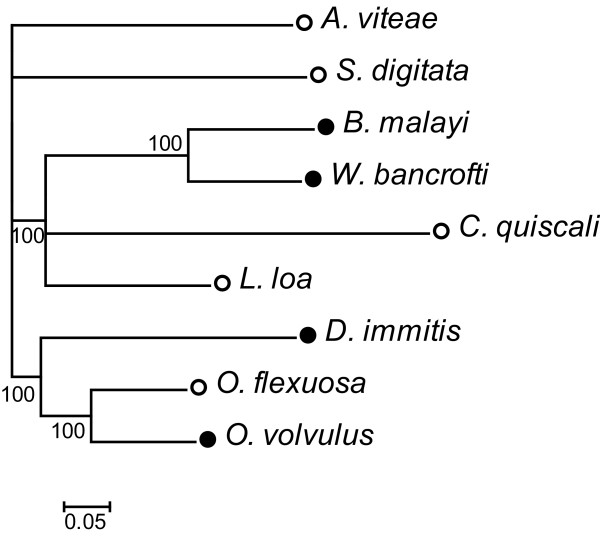
**Phylogeny of filarial nematodes based on mtDNA sequences.** Phylogenetic analysis was based on the concatenated nucleotide sequences of the twelve protein coding genes. Percentages of Bayesian posterior probabilities are displayed at nodes. Black circles indicate *Wolbachia*-dependence while white circles indicate *Wolbachia*-independence.

A phylogenetic analysis was carried out using the nucleotide sequences of the 12 protein coding genes from the fully-sequenced filarial mtDNAs (Figure 4). Trees were left unrooted since the closest relatives of filarial nematodes with complete mtDNA sequences (i.e. *Ascaris* and *Toxocara* species) are still too divergent to allow for accurate alignment. Overall, topology is similar to that of trees based on single mitochondrial genes (i.e. 12s rRNA gene, Cox I) with improved statistical support [2,26,30]. As in the previous studies, our molecular phylogeny does not agree with the classical taxonomy of the filariae, as the Dirofilariinae and Onchocercinae appear as polyphyletic groups. In our tree, the lymphatic filariae cluster together, as do the genera *Onchocerca* and *Dirofilaria*. *C. quiscali*, which has not been included in previous analyses, is most closely related to the lymphatic filariae and *L. loa*. *A. viteae* and *S. digitata* are basal to the other species in our study. Simpler neighbor joining trees were constructed for each individual gene (data not shown). As in the tree based on concatenated protein coding sequences, clustering is never reflective of *Wolbachia* status.

## Discussion

Filarial nematodes are widespread parasites that infect all classes of vertebrates except fish [31]. Many of these are of socioeconomic and medical importance. However, the mtDNAs had only been sequenced from 4 filarial species [22-25]. In our study we characterized the mtDNAs of 5 additional species and used the newly available sequences to compare the mtDNA of *Wolbachia*-dependent and independent filarial nematodes. Initially, we hypothesized that there might be obvious differences in the mitochondrial genome sequences of *Wolbachia*-dependent and independent species due to the evolutionary pressure exerted by a co-transmitted endosymbiont that may impact the energy balance of its host [14,16-21]. However, our data indicate that the mtDNAs of filarial nematodes are, thus far, remarkably similar. No major differences in genome length, AT content or codon usage were detected. All 9 species contain the standard 12 protein-coding genes, 22 tRNAs and 2 rRNAs. Differences in tRNA structure or anticodon usage are also minor and do not correlate to *Wolbachia* status. In light of these findings, it seems that *Wolbachia* has had little effect on the content of filarial mtDNA.

In most filarial species, synteny was perfectly preserved. The rearrangement of 5 tRNA’s in *C. quiscali* probably reflects its evolutionary distance from the other species included in this study rather than its *Wolbachia*-free status (see Figure 4), as such rearrangements were not detected in other *Wolbachia*-free species. It will be necessary to sequence the mtDNAs of other members of the Splendidofilariinae in order to determine whether this rearrangement is species-specific or typical of the entire subfamily. However, this minor alteration in gene order is unlikely to impact overall mitochondrial function.

If *Wolbachia* infection had led to the fixation of certain mitochondrial types in an ancestral population, one might expect to see higher degrees of sequence identity between species that have come into contact with the endosymbiont (i.e. species from the Onchocercinae and Dirofilariinae) as compared to species that have not [2]. However, our phylogenetic analysis indicated that the level of sequence identity shared between mitochondrial protein-coding genes is independent of *Wolbachia* status. If it is true that filarial species considered primitive based on classical taxonomy (i.e. *C. quiscali* and *S. digitata*) were never associated with *Wolbachia*, the similarity of their mtDNAs to the others in this study makes it seem unlikely that *Wolbachia* have had a significant impact on mtDNA sequence. The lack of diversity in mtDNA sequences could be taken as an indication that the initial infection and theoretical *Wolbachia*-induced genetic bottleneck occurred in an ancestor of all filarial nematodes, but this is unlikely given the phylogenetic age of the filariae. Of course, one must consider that this study was performed on the level of complete genes. In the future, it may be informative to compare smaller loci in *Wolbachia*-dependent and independent species, as even single base changes are known to have profound effects on mitochondrial function [32].

The fact that no sequence-level differences were observed between mitochondria from *Wolbachia*-dependent and independent filarial species does not exclude the possibility that differences may exist in mitochondrial function or efficiency between these groups. Of course, many of the genes related to oxidative phosphorylation and energy metabolism are encoded in the nuclear genome rather than the mitochondrial genome. Because only one filarial genome has been published (see [24] for the genome of *B. malayi*), we do not know if certain nuclear genes are missing or altered in *Wolbachia*-dependent species relative to their *Wolbachia*-independent counterparts.

Even if there are no differences in the genes encoded by *Wolbachia*-dependent and independent filarial worms, it is possible that variation in expression levels could lead to differences in mitochondrial output. Expression levels may be partially dictated by the number of mitochondrial genomes present in each mitochondria or the density of mitochondria in a given organism. These factors are also variable. Quantitative real-time PCR techniques could be used to assess expression; however, careful normalization would be necessary to ensure accurate results when comparing expression levels across multiple species and life cycle stages. This type of analysis will not be possible until better nuclear genome data is produced to provide appropriate control sequences.

Mitochondria and *Wolbachia* appear to share a common evolutionary story wherein bacteria of the Rickettsial family were taken up and transformed over time into an obligate mutualist that provides products essential to the life of the host. Since *Wolbachia* and mitochondria are co-transmitted and since *Wolbachia* may have an impact on host energy metabolism [14], it is possible that *Wolbachia* have affected the mitochondria of *Wolbachia*-dependent species in ways that are not reflected in the mtDNA sequence. Additional research will be required to test this hypothesis.

## Conclusions

Here we report the mitochondrial genome sequences of 5 species of filarial nematodes: *Acanthocheilonema viteae, Chandlerella quiscali*, *Loa loa*, *Onchocerca flexuosa*, and *Wuchereria bancrofti*. Although we had hypothesized that the presence of *Wolbachia* endobacteria in some filarial nematodes may have had an impact on the content, organization or sequence of filarial mtDNA, we found no evidence that supports this hypothesis. The 9 available filarial mitochondrial sequences are remarkably similar on the sequence level. Future studies may determine whether functional differences exist between the mitochondria of *Wolbachia*-dependent and independent filarial nematodes.

## Methods

### Parasite materials

Adult *B. malayi* and *A. viteae* were obtained from experimentally infected Mongolian jirds as previously described [33,34]. Adult *D. immitis* were obtained from the Filariasis Research Reagent Resource Center (Athens, GA). Adult *O. flexuosa* were isolated from subcutaneous nodules dissected from red deer (*Cervus elaphus*) in Schleswig-Holstein, Germany [10]. Adult *O. volvulus*, and microfilariae of *W. bancrofti* and *L. loa* were available from prior studies in Uganda, Papua New Guinea and Cameroon, respectively [8,35,36]. Adult *Chandlerella quiscali* were obtained from common grackles *Quiscalus quiscula* trapped in North Dakota, USA.

### Nucleic acid isolation and cDNA synthesis

DNA for sequencing was isolated from adult worms and microfilariae using the DNeasy Blood and Tissue Kit (Qiagen, Valencia, CA), ethanol precipitated to concentrate and stored in 1x TE buffer. RNA was isolated as previously described [29]. Briefly, worms were homogenized by bead-beating in TRIzol (Invitrogen, Carlsbad, CA) and RNA was isolated by organic extraction with 1-bromo-3-chloropropane followed by column purification using the RNeasy Mini Kit (Qiagen) including an on-column DNase digest. A second DNase treatment was performed with the TURBO DNA-*free* Kit (Applied Biosystems, Austin, TX). cDNA was synthesized from total RNA using qScript cDNA SuperMix according to manufacturer’s suggested protocol (Quant Biosciences, Gaithersburg, MD) and purified with the Qiaquick PCR Purification Kit (Qiagen).

### PCR reactions and sequencing

Primers used to amplify mtDNA in 10 segments are reported in Additional file 2: Table S2. “Filarial Mito” primer sets are designed to target conserved portions of filarial mitochondria. In cases where the conserved primer set failed, species-specific primer sets were implemented. This was the case for segments 1–2, 7 and 9 in *A. viteae*, segments 5 and 8 in *C. quiscali*, segments 1, 4, 8 and 10 in *L. loa,* and segments 7 and 9 in *W. bancrofti*. All PCR reactions were performed using the Platinum Taq High Fidelity DNA polymerase (Invitrogen) according to the manufacturer’s suggested protocol with annealing temperatures adjusted to accommodate the thermodynamic properties of the primers. PCR products were cloned using the TOPO-TA Cloning Kit for Sequencing or the TOPO-XL PCR Cloning Kit (Invitrogen) depending on size, and sequenced by primer walking.

Species-specific primers (given “RT” designation in the primer name) were designed to detect protein-coding sequences from cDNA. The sequences of these primers are reported in Additional file 2: Table S2. To detect expression, each PCR reaction included a DNA positive control, a cDNA test sample and total RNA and water-only negative controls.

### Assembly and annotation of the mitochondrial genomes

Contigs were assembled using Contig Express and analyzed using Vector NTI version 10.3.1 (Invitrogen). Sequences were verified by comparison with publically available sequence data from the Genbank sequence read archive for *L. loa* (accession number SRP000756) and *W. bancrofti* (accession number SRP000772).

Protein-coding genes (including initiation and termination codons) and rRNAs were determined based on their homology to sequences reported from the mitochondrial genomes of *B. malayi, D. immitis, O. volvulus* and *S. digitata*[22-25].

In most instances, tRNA sequences were predicted using Arwen (available at http://130.235.46.10/ARWEN/) [37] and verified by homology to known filarial tRNA sequences. Any computationally predicted tRNAs that fell within other documented structures (i.e. protein-coding genes or rRNAs) were disregarded. tRNA^Ala^ and tRNA^Leu2^ in *A. viteae*, tRNA^Leu2^ and tRNA^Gly^ in *O. flexuosa*, and tRNA^Ala^ in *L. loa* were identified solely based on homology to known orthologs and the presence of the expected anticodon.

Base composition and codon usage were calculated using the DNA Stats and codon usage features available from the Sequence Manipulation Suite (http://www.bioinformatics.org/sms2/dna_stats.html). Diagrams of complete mtDNAs were constructed using DNA plotter (http://www.sanger.ac.uk/resources/software/dnaplotter/) [38].

### Phylogenetic analysis

The nucleotide sequences of the 12 protein coding genes, excluding stop codons, were aligned using Clustal W as implemented in MEGA4 using default parameters [39]. Individual gene alignments were concatenated using FASconCAT [40].

Model selection was performed using MrModeltest2.3 according to the Akaike information criterion [41]. Bayesian Metropolis-coupled Markov chain Monte Carlo (MCMC) analysis was performed on the dataset with the GTR + I + G nucleotide substitution model by MrBayes Version 3.1.2 [42,43]. Two simultaneous runs of 500,000 generations were performed with sampling every 100 generations and a 25% burn-in. The resulting phylogenetic tree was visualized in MEGA4 [39].

## Abbreviations

ATP6, ATP synthase subunit 6; CytB, Cytochrome b; Cox1, Cox2 and Cox3, cytochrome C oxidase subunits 1–3; ND1–6, and NDL4, Nicotinamide adenine dinucleotide dehydrogenase subunits 1–6, and L4; rRNA, Ribosomal RNA; tRNA, Transfer RNA.

## Competing interests

The authors declare that they have no competing interests.

## Authors’ contributions

SNM sequenced and assembled the mitochondrial genomes, carried out bioinformatic analyses and drafted the manuscript. ASM assisted in sequencing and assembling the mitochondrial genomes. JAV and VVT collected parasite material and assisted in revising the manuscript. GJW and PUF supervised study design and assisted in drafting and revising the manuscript. All authors have read and approved the final manuscript.

## Supplementary Material

Additional file 1: Table S1. Codon usage in the mitochondrial genomes of filarial nematodes Click here for file

Additional file 2: Table S2. Primer sequences Click here for file

## References

[B1] Bandi C Anderson TJ Genchi C Blaxter ML Phylogeny ofWolbachiain filarial nematodes Proc Biol Sci 1998 265 1413 2407 2413 10.1098/rspb.1998.0591 9921679 PMC1689538

[B2] Ferri E Bain O Barbuto M Martin C Lo N Uni S Landmann F Baccei SG Guerrero R de Souza Lima S Bandi C Wanji S Diagne M Casiraghi M New Insights into the Evolution ofWolbachiaInfections in Filarial Nematodes Inferred from a Large Range of Screened Species PLoS One 2011 6 6 e20843 10.1371/journal.pone.0020843 21731626 PMC3120775

[B3] Kozek WJ Marroquin HF Intracytoplasmic bacteria inOnchocerca volvulus AmJTrop Med Hyg 1977 26 4 663 678 10.4269/ajtmh.1977.26.663 889009

[B4] Vincent AL Ash LR Frommes SP The ultrastructure of adultBrugia malayi(Brug, 1927) (Nematoda: Filarioidea) J Parasitol 1975 61 3 499 512 10.2307/3279332 1138041

[B5] Hoerauf A Mand S Fischer K Kruppa T Marfo-Debrekyei Y Debrah AY Pfarr KM Adjei O Buttner DW Doxycycline as a novel strategy against bancroftian filariasis-depletion ofWolbachiaendosymbionts fromWuchereria bancroftiand stop of microfilaria production Med Microbiol Immunol 2003 192 4 211 216 10.1007/s00430-002-0174-6 12684759

[B6] Hoerauf A Mand S Volkmann L Buttner M Marfo-Debrekyei Y Taylor M Adjei O Buttner DW Doxycycline in the treatment of human onchocerciasis: Kinetics ofWolbachiaendobacteria reduction and of inhibition of embryogenesis in femaleOnchocercaworms Microbes Infect 2003 5 4 261 273 10.1016/S1286-4579(03)00026-1 12706439

[B7] Hoerauf A Nissen-Pahle K Schmetz C Henkle-Duhrsen K Blaxter ML Buttner DW Gallin MY Al-Qaoud KM Lucius R Fleischer B Tetracycline therapy targets intracellular bacteria in the filarial nematodeLitomosoides sigmodontisand results in filarial infertility J Clin Invest 1999 103 1 11 18 10.1172/JCI4768 9884329 PMC407866

[B8] Buttner DW Wanji S Bazzocchi C Bain O Fischer P Obligatory symbioticWolbachiaendobacteria are absent fromLoa loa Filaria J 2003 2 1 10 10.1186/1475-2883-2-10 12801420 PMC161789

[B9] McNulty SN Fischer K Mehus JO Vaughan JA Tkach VV Weil GJ Fischer PU Absence of Wolbachia Endobacteria in Chandlerella quiscali , an Avian Filarial Parasite J Parasitol 2012 98 2 382 387 10.1645/GE-2879.1 22032328 PMC5000782

[B10] Plenge-Bonig A Kromer M Buttner DW Light and electron microscopy studies onOnchocerca jakutensisandO. flexuosaof red deer show different host-parasite interactions Parasitol Res 1995 81 1 66 73 10.1007/BF00932419 7724516

[B11] Andersson SG Zomorodipour A Andersson JO Sicheritz-Ponten T Alsmark UC Podowski RM Naslund AK Eriksson AS Winkler HH Kurland CG The genome sequence ofRickettsia prowazekiiand the origin of mitochondria Nature 1998 396 6707 133 140 10.1038/24094 9823893

[B12] Dumler JS Barbet AF Bekker CP Dasch GA Palmer GH Ray SC Rikihisa Y Rurangirwa FR Reorganization of genera in the families Rickettsiaceae and Anaplasmataceae in the order Rickettsiales: unification of some species ofEhrlichiawithAnaplasma,CowdriawithEhrlichiaandEhrlichiawithNeorickettsia, descriptions of six new species combinations and designation ofEhrlichia equiand ‘HGE agent’ as subjective synonyms ofEhrlichia phagocytophila Int J Syst Evol Microbiol 2001 51 Pt 6 2145 2165 11760958 10.1099/00207713-51-6-2145

[B13] Foster J Ganatra M Kamal I Ware J Makarova K Ivanova N Bhattacharyya A Kapatral V Kumar S Posfai J Vincze T Ingram J Moran L Lapidus A Omelchenko M Kyrpides N Ghedin E Wang S Goltsman E Joukov V Ostrovskaya O Tsukerman K Mazur M Comb D Koonin E Slatko B TheWolbachiagenome ofBrugia malayi: endosymbiont evolution within a human pathogenic nematode PLoS Biol 2005 3 4 e121 10.1371/journal.pbio.0030121 15780005 PMC1069646

[B14] Strubing U Lucius R Hoerauf A Pfarr KM Mitochondrial genes for heme-dependent respiratory chain complexes are up-regulated after depletion ofWolbachiafrom filarial nematodes Int J Parasitol 2010 40 10 1193 1202 10.1016/j.ijpara.2010.03.004 20362581

[B15] Werren JH Baldo L Clark ME Wolbachia: master manipulators of invertebrate biology Nat Rev Microbiol 2008 6 10 741 751 10.1038/nrmicro1969 18794912

[B16] Delgado AM Cook JM Effects of a sex-ratio distorting endosymbiont on mtDNA variation in a global insect pest BMC Evol Biol 2009 9 49 10.1186/1471-2148-9-49 19257899 PMC2671496

[B17] Hurst GD Jiggins FM Problems with mitochondrial DNA as a marker in population, phylogeographic and phylogenetic studies: the effects of inherited symbionts Proc Biol Sci 2005 272 1572 1525 1534 10.1098/rspb.2005.3056 16048766 PMC1559843

[B18] Raychoudhury R Grillenberger BK Gadau J Bijlsma R van de Zande L Werren JH Beukeboom LW Phylogeography ofNasonia vitripennis(Hymenoptera) indicates a mitochondrial-Wolbachiasweep in North America Heredity 2010 104 3 318 326 10.1038/hdy.2009.160 20087396

[B19] Turelli M Hoffmann AA Rapid spread of an inherited incompatibility factor in CaliforniaDrosophila Nature 1991 353 6343 440 442 10.1038/353440a0 1896086

[B20] Turelli M Hoffmann AA McKechnie SW Dynamics of cytoplasmic incompatibility and mtDNA variation in naturalDrosophila simulanspopulations Genetics 1992 132 3 713 723 1468627 10.1093/genetics/132.3.713 PMC1205209

[B21] Belanger DH Perkins SL Wolbachiainfection and mitochondrial diversity in the canine heartworm (Dirofilaria immitis) Mitochondrial DNA 2010 21 6 227 233 10.3109/19401736.2010.533765 21171866

[B22] Keddie EM Higazi T Unnasch TR The mitochondrial genome ofOnchocerca volvulus: sequence, structure and phylogenetic analysis Mol Biochem Parasitol 1998 95 1 111 127 10.1016/S0166-6851(98)00102-9 9763293

[B23] Hu M Gasser RB Abs El-Osta YG Chilton NB Structure and organization of the mitochondrial genome of the canine heartworm,Dirofilaria immitis Parasitology 2003 127 Pt 1 37 51 12885187 10.1017/s0031182003003275

[B24] Ghedin E Wang S Spiro D Caler E Zhao Q Crabtree J Allen JE Delcher AL Guiliano DB Miranda-Saavedra D Angiuoli SV Creasy T Amedeo P Haas B El-Sayed NM Wortman JR Feldblyum T Tallon L Schatz M Shumway M Koo H Salzberg SL Schobel S Pertea M Pop M White O Barton GJ Carlow CK Crawford MJ Daub J Dimmic MW Estes CF Foster JM Ganatra M Gregory WF Johnson NM Jin J Komuniecki R Korf I Kumar S Laney S Li BW Li W Lindblom TH Lustigman S Ma D Maina CV Martin DM McCarter JP McReynolds L Mitreva M Nutman TB Parkinson J Peregrin-Alvarez JM Poole C Ren Q Saunders L Sluder AE Smith K Stanke M Unnasch TR Ware J Wei AD Weil G Williams DJ Zhang Y Williams SA Fraser-Liggett C Slatko B Blaxter ML Scott AL Draft genome of the filarial nematode parasiteBrugia malayi Science 2007 317 5845 1756 1760 10.1126/science.1145406 17885136 PMC2613796

[B25] Yatawara L Wickramasinghe S Rajapakse RP Agatsuma T The complete mitochondrial genome ofSetaria digitata(Nematoda: Filarioidea): Mitochondrial gene content, arrangement and composition compared with other nematodes Mol Biochem Parasitol 2010 173 1 32 38 10.1016/j.molbiopara.2010.05.004 20470833

[B26] Casiraghi M Bain O Guerrero R Martin C Pocacqua V Gardner SL Franceschi A Bandi C Mapping the presence ofWolbachia pipientison the phylogeny of filarial nematodes: evidence for symbiont loss during evolution Int J Parasitol 2004 34 2 191 203 10.1016/j.ijpara.2003.10.004 15037105

[B27] McLaren DJ Worms MJ Laurence BR Simpson MG Micro-organisms in filarial larvae (Nematoda) Trans R Soc Trop Med Hyg 1975 69 5–6 509 514 1228988 10.1016/0035-9203(75)90110-8

[B28] McGarry HF Pfarr K Egerton G Hoerauf A Akue JP Enyong P Wanji S Klager SL Bianco AE Beeching NJ Taylor MJ Evidence againstWolbachiasymbiosis inLoa loa Filaria J 2003 2 1 9 10.1186/1475-2883-2-9 12816546 PMC161820

[B29] McNulty SN Foster JM Mitreva M Dunning Hotopp JC Martin J Fischer K Wu B Davis PJ Kumar S Brattig NW Slatko BE Weil GJ Fischer PU Endosymbiont DNA in endobacteria-free filarial nematodes indicates ancient horizontal genetic transfer PLoS One 2010 5 6 e11029 10.1371/journal.pone.0011029 20543958 PMC2882956

[B30] Casiraghi M Anderson TJ Bandi C Bazzocchi C Genchi C A phylogenetic analysis of filarial nematodes: comparison with the phylogeny ofWolbachiaendosymbionts Parasitology 2001 122 Pt 1 93 103 11197770 10.1017/s0031182000007149

[B31] Anderson RC Bain O Anderson RC, Chabaud AG, Willmott S Keys to genera of the order Spirurida. Part 3. Diplotriaenoidea, Aproctoidea and Filarioidea In CIH keys to the nematode parasites of vertebrates 1976 Commonwealth Agricultural Bureau, Farnham Royal, UK 59 116

[B32] Wong LJ Molecular genetics of mitochondrial disorders Dev Disabil Res Rev 2010 16 2 154 162 10.1002/ddrr.104 20818730

[B33] Ash LR Riley JM Development of subperiodicBrugia malayiin the jird,Meriones unguiculatus, with notes on infections in other rodents J Parasitol 1970 56 5 969 973 10.2307/3277515 5504534

[B34] Lucius R Textor G Acanthocheilonema viteae: rational design of the life cycle to increase production of parasite material using less experimental animals Appl Parasitol 1995 36 1 22 33 7780447

[B35] Fischer P Kipp W Bamuhiga J Binta-Kahwa J Kiefer A Buttner DW Parasitological and clinical characterization ofSimulium neavei-transmitted onchocerciasis in western Uganda Trop Med Parasitol 1993 44 4 311 321 8134773

[B36] Weil GJ Kastens W Susapu M Laney SJ Williams SA King CL Kazura JW Bockarie MJ The impact of repeated rounds of mass drug administration with diethylcarbamazine plus albendazole on bancroftian filariasis in Papua New Guinea PLoS Negl Trop Dis 2008 2 12 e344 10.1371/journal.pntd.0000344 19065257 PMC2586652

[B37] Laslett D Canback B ARWEN: a program to detect tRNA genes in metazoan mitochondrial nucleotide sequences Bioinformatics 2008 24 2 172 175 10.1093/bioinformatics/btm573 18033792

[B38] Carver T Thomson N Bleasby A Berriman M Parkhill J DNAPlotter: circular and linear interactive genome visualization Bioinformatics 2009 25 1 119 120 10.1093/bioinformatics/btn578 18990721 PMC2612626

[B39] Tamura K Dudley J Nei M Kumar S MEGA4: Molecular Evolutionary Genetics Analysis (MEGA) software version 4.0 Mol Biol Evol 2007 24 8 1596 1599 10.1093/molbev/msm092 17488738

[B40] Kuck P Meusemann K FASconCAT: Convenient handling of data matrices Mol Phylogenet Evol 2010 56 3 1115 1118 10.1016/j.ympev.2010.04.024 20416383

[B41] Nylander JAA MrModeltest v2. 2004, Program distributed by the author 2004 Evolutionary Biology Centre, Uppsala University,

[B42] Huelsenbeck JP Ronquist F MRBAYES: Bayesian inference of phylogenetic trees Bioinformatics 2001 17 8 754 755 10.1093/bioinformatics/17.8.754 11524383

[B43] Ronquist F Huelsenbeck JP MrBayes 3: Bayesian phylogenetic inference under mixed models Bioinformatics 2003 19 12 1572 1574 10.1093/bioinformatics/btg180 12912839

