# Supplementary material for: Comparing the mitochondrial genomes of Wolbachia-dependent and independent filarial nematode species
Source: BMC Genomics. 2012 Apr 24;13:145. doi: 10.1186/1471-2164-13-145 (PMC3409033; doi:10.1186/1471-2164-13-145)
Supplement: Additional file 1: Table S1. — Codon usage in the mitochondrial genomes of filarial nematodes [file 1471-2164-13-145-S1.docx]

**Table S1. Codon usage in the mitochondrial genomes of filarial nematodes**

| Amino Acid | Codon | *Wolbachia*-dependent | | | | *Wolbachia-*independent, *Wolbachia*-associated subfamiliy | | | *Wolbachia*-independent, *Wolbachia*-unassociated subfamily | |
| --- | --- | --- | --- | --- | --- | --- | --- | --- | --- | --- |
|  |  | *B. malayi* | *D. immitis* | *O. volvulus* | *W. bancrofti* | *A. viteae* | *L. loa* | *O. flexuosa* | *C. quiscali* | *S. digitata* |
| Ala | GCG | 4 | 6 | 6 | 2 | 6 | 4 | 1 | 1 | 0 |
| Ala | GCA | 7 | 1 | 2 | 3 | 0 | 1 | 5 | 5 | 0 |
| Ala | GCT | 58 | 61 | 72 | 73 | 65 | 74 | 82 | 51 | 76 |
| Ala | GCC | 12 | 4 | 5 | 5 | 4 | 2 | 0 | 0 | 0 |
| Cys | TGT | 96 | 103 | 97 | 97 | 100 | 101 | 92 | 88 | 95 |
| Cys | TGC | 10 | 4 | 3 | 6 | 5 | 3 | 4 | 1 | 2 |
| Asp | GAT | 79 | 90 | 82 | 81 | 80 | 84 | 80 | 80 | 84 |
| Asp | GAC | 8 | 2 | 1 | 5 | 2 | 1 | 4 | 1 | 2 |
| Glu | GAG | 30 | 46 | 45 | 39 | 50 | 26 | 45 | 20 | 39 |
| Glu | GAA | 28 | 14 | 21 | 22 | 12 | 35 | 18 | 45 | 21 |
| Phe | TTT | 615 | 656 | 620 | 618 | 622 | 616 | 604 | 647 | 645 |
| Phe | TTC | 15 | 2 | 5 | 14 | 7 | 3 | 12 | 13 | 3 |
| Gly | GGG | 27 | 45 | 29 | 25 | 44 | 13 | 25 | 9 | 16 |
| Gly | GGA | 26 | 25 | 20 | 25 | 22 | 30 | 22 | 38 | 10 |
| Gly | GGT | 156 | 155 | 185 | 173 | 162 | 177 | 187 | 173 | 204 |
| Gly | GGC | 25 | 12 | 11 | 8 | 14 | 6 | 6 | 2 | 9 |
| His | CAT | 48 | 52 | 52 | 48 | 53 | 49 | 53 | 51 | 52 |
| His | CAC | 4 | 1 | 2 | 4 | 0 | 4 | 1 | 2 | 1 |
| Ile | ATT | 223 | 198 | 191 | 195 | 198 | 221 | 198 | 251 | 195 |
| Ile | ATC | 13 | 1 | 2 | 6 | 3 | 5 | 2 | 5 | 6 |
| Lys | AAG | 43 | 57 | 69 | 51 | 59 | 54 | 69 | 43 | 65 |
| Lys | AAA | 28 | 19 | 8 | 21 | 10 | 21 | 7 | 31 | 7 |
| Leu | TTG | 182 | 297 | 303 | 241 | 253 | 246 | 266 | 191 | 266 |
| Leu | TTA | 224 | 120 | 139 | 180 | 131 | 179 | 164 | 222 | 144 |
| Leu | CTG | 12 | 5 | 6 | 2 | 9 | 1 | 8 | 2 | 1 |
| Leu | CTA | 13 | 2 | 1 | 7 | 3 | 4 | 3 | 4 | 1 |
| Leu | CTT | 25 | 25 | 26 | 30 | 35 | 26 | 33 | 24 | 31 |
| Leu | CTC | 2 | 1 | 2 | 0 | 3 | 0 | 0 | 0 | 4 |
| Met | ATG | 73 | 103 | 112 | 88 | 101 | 88 | 89 | 64 | 91 |
| Met | ATA | 74 | 39 | 29 | 61 | 54 | 58 | 69 | 91 | 52 |
| Asn | AAT | 97 | 90 | 80 | 93 | 83 | 102 | 89 | 107 | 96 |
| Asn | AAC | 5 | 3 | 6 | 9 | 4 | 4 | 4 | 2 | 4 |
| Pro | CCG | 2 | 4 | 5 | 3 | 2 | 0 | 3 | 3 | 2 |
| Pro | CCA | 11 | 1 | 3 | 7 | 9 | 5 | 5 | 10 | 1 |
| Pro | CCT | 58 | 68 | 66 | 66 | 61 | 70 | 68 | 61 | 75 |
| Pro | CCC | 4 | 1 | 2 | 1 | 4 | 0 | 3 | 2 | 1 |
| Gln | CAG | 26 | 42 | 33 | 30 | 30 | 32 | 33 | 14 | 37 |
| Gln | CAA | 25 | 9 | 17 | 22 | 18 | 17 | 18 | 31 | 16 |
| Arg | CGG | 7 | 7 | 10 | 6 | 4 | 0 | 2 | 3 | 0 |
| Arg | CGA | 3 | 5 | 0 | 2 | 4 | 0 | 3 | 2 | 3 |
| Arg | CGT | 42 | 43 | 46 | 45 | 47 | 55 | 50 | 51 | 52 |
| Arg | CGC | 2 | 0 | 0 | 1 | 2 | 0 | 1 | 0 | 1 |
| Ser | AGG | 13 | 17 | 27 | 15 | 22 | 7 | 8 | 16 | 5 |
| Ser | AGA | 24 | 15 | 13 | 19 | 16 | 20 | 26 | 34 | 6 |
| Ser | AGT | 113 | 117 | 116 | 121 | 114 | 128 | 123 | 102 | 141 |
| Ser | AGC | 9 | 3 | 1 | 1 | 4 | 2 | 6 | 0 | 2 |
| Ser | TCG | 1 | 8 | 1 | 2 | 4 | 1 | 3 | 1 | 1 |
| Ser | TCA | 5 | 8 | 4 | 9 | 5 | 6 | 5 | 11 | 3 |
| Ser | TCT | 161 | 162 | 162 | 149 | 162 | 171 | 158 | 169 | 173 |
| Ser | TCC | 3 | 1 | 3 | 10 | 5 | 2 | 2 | 2 | 3 |
| Thr | ACG | 0 | 2 | 4 | 0 | 1 | 1 | 0 | 3 | 2 |
| Thr | ACA | 4 | 2 | 0 | 2 | 2 | 3 | 5 | 7 | 0 |
| Thr | ACT | 84 | 88 | 85 | 82 | 90 | 82 | 88 | 66 | 81 |
| Thr | ACC | 3 | 0 | 0 | 5 | 0 | 2 | 2 | 2 | 0 |
| Val | GTG | 19 | 40 | 41 | 20 | 39 | 24 | 21 | 10 | 22 |
| Val | GTA | 26 | 19 | 27 | 28 | 20 | 26 | 25 | 40 | 12 |
| Val | GTT | 221 | 248 | 256 | 265 | 263 | 248 | 244 | 221 | 273 |
| Val | GTC | 17 | 2 | 1 | 6 | 7 | 4 | 8 | 2 | 3 |
| Trp | TGG | 35 | 41 | 46 | 45 | 47 | 30 | 39 | 23 | 48 |
| Trp | TGA | 39 | 32 | 29 | 33 | 32 | 45 | 36 | 51 | 29 |
| Tyr | TAT | 214 | 230 | 223 | 216 | 239 | 232 | 223 | 243 | 243 |
| Tyr | TAC | 15 | 1 | 4 | 14 | 8 | 1 | 9 | 3 | 0 |
| End | TAG | 4 | 5 | 7 | 2 | 3 | 3 | 3 | 4 | 5 |
| End | TAA | 8 | 7 | 5 | 10 | 9 | 9 | 9 | 8 | 7 |
