# Supplementary material for: Comparing the mitochondrial genomes of Wolbachia-dependent and independent filarial nematode species
Source: BMC Genomics. 2012 Apr 24;13:145. doi: 10.1186/1471-2164-13-145 (PMC3409033; doi:10.1186/1471-2164-13-145)
Supplement: Additional file 2: Table S2. — Primer sequences [file 1471-2164-13-145-S2.docx]

**Table S2. Primer sequences**

| **Primer Set** | **Forward Primer (5’-3’)** | **Reverse Primer (5’-3’)** |
| --- | --- | --- |
| Filarial Mito #1 | ttatttttaattttcgatta | acactcataaaagacaaaa |
| Filarial Mito #2 | tttttaatgttcctttgagtattactttt | tagcaatatgataaaactcaccaataaa |
| Filarial Mito #3 | ttgtaagtctttggctgctta | tcataaaaagaagtattaaaattacgat |
| Filarial Mito #4 | tgttgggtgctattaatttt | ctacaaaaataagttaacaaagaaa |
| Filarial Mito #5 | gtcgtgttggttatggttatca | aataaggaacagaagttatcaaactagtaat |
| Filarial Mito #6 | tctaatggggcttctatgt | gaacctcaaacatataataaacaa |
| Filarial Mito #7 | ttaattggtatcatattcaggcttat | aattttgattacctgggtactaatc |
| Filarial Mito #8 | agatagtattacttatttttagttt | aaatataactctgcaaaaa |
| Filarial Mito #9 | catgttggttttaatttggt | aaaaactactaacacccaaaaa |
| Filarial Mito #10 | ttctcgtttgttttattttt | aagtaatactcaaaggaacatt |
| Av Mito #1-2 | tgctcggtttgtttataagtttgttcctg | caaacagaactaaaccaatttaacatcaaatactca |
| Av Mito #7 | gccatattttgaagtattctcgtcgtttt | gagggctatcctagcacatattccaaa |
| Av Mito #9 | ggccatgggcttgtattggttt | ggagccgccatagccttagga |
| Cq Mito #5 | ggaaggaggttatcgtcgtgttgg | cgggaaccacatgtgcagga |
| Cq Mito #8 | tttttggtgttgtttctggggttg | tctttaactgaaaaataaatctaaatgacccaaaca |
| Ll Mito #4 | tggcttttcctcgtttgaatgc | ccaaatatggacacccaaagaaatataaca |
| Ll Mito #8 | tgttaagggttttggtattgataaggttatgtttt | caaaaccaaatcgcagacaacgaat |
| Ll Mito #10-1 | tgtataattcgttgtctgcgatttgg | cagaaacacaaataaaaccaagtacaaaaacact |
| Wb Mito #7 | tcgtcgatatttgtgtttaaatatggctaagtgtg | gggctctccatacgcatgttcca |
| Wb Mito #9 | cctttgtttagtccttgagctagtgttgg | caggagtaggagcagccatagcc |
| Av 1ND2 RT | tgtgctggtatgagggggagtt | gcccagacccaaacaacaaca |
| Av 2ND4 RT | gcccaaggtccatgtggagt | ggcaaacaaacaacactacgacca |
| Av 3Cox1 RT | gccgggttggatgtcgatac | ccctgtatgccagcaaaatgc |
| Av 4ND6 RT | gggtgcatgtgtggtattc | agaaaaatcaaaaccaaaagaaa |
| Av 5CytB RT | gaggtattgggctgcggttg | tgaccagcccaagtcaatcaaa |
| Av 6Cox3 RT | gggctgggttggtaggttttg | catgaaaaccatggagaccagtacc |
| Av 7NDL4 RT | tgatcgattgatttttgttatgttga | cataaccttatccacaccaaaacc |
| Av8 ND1 RT | tgtgggctgtttcaagctttgt | tcagaaaaatcaaaaggagcacga |
| Av 9ATP6 RT | ggttttaatcatcaaggatttcagtc | caaaaacatacaaaccagagtcaaaa |
| Av 10Cox2 RT | agggcggatatcccgtcagt | acggatcaccccttccaaca |
| Av 11ND3 RT | tttgattgtgttggttttggtt | ccataataccactccatataaaaactca |
| Av 12ND5 RT | tggcggctcctactcctgtt | ggaccaaaattccccactcg |
| Bm 1ND2 RT | tggatctgctccttttcatttttga | aacccaaccatactagaagtgccaaa |
| Bm 2ND4 RT | caggttgagaagatcagggcttg | cggtaaataccggctccacct |
| Bm 3Cox1 RT | tgcagccgttatgatgctga | ccctgtatgccagcaaaatgc |
| Bm 4ND6 RT | tttctttgggtgttcatgtgtga | caaaccacgtataaacctatcacaacc |
| Bm 5CytB RT | gcccatgtggtccctgaatg | cggcccaagtcaaccaaaaa |
| Bm 6Cox3 RT | cttggttgggtggggtttga | tccatgcaaaccagttccgata |
| Bm 7NDL4 RT | atctaatttttgttttgctt | aacaccaaaacttttaacac |
| Bm8 ND1 RT | ggtggtttacgggcttgtgc | aaatcgaaaggggcacgatg |
| Bm 9ATP6 RT | ggttttagccatcagggtgttca | cgcaaagttaaagccacaccac |
| Bm 10Cox2 RT | tggacctgggttttggttga | caactccaaagcaatgggcata |
| Bm 11ND3 RT | tctttgagggttcgggatag | aacttcccaaaataccattcca |
| Bm 12ND5 RT | tgcagggtgcttctcgtttg | ggcaaccaactgccaaaagg |
| Di 1ND2 RT | tttgggtgtttagagttttgggtggt | tcaaaccctgattaaaccccgaca |
| Di 2ND4 RT | gggattgtaagtctttggcggctta | accccgcccatcaaacacat |
| Di 3Cox1 RT | ggtgcccctgaaatggcttt | ccagccaaaacaggcactga |
| Di 4ND6 RT | tgaggtttttagagtgggattctttga | aagaatatcaaacatgaatgccaaaaga |
| Di 5CytB RT | gggggcttttgcttctgttca | ccttcaaccaatagcccggaaa |
| Di 6Cox3 RT | tggattctgctttgggtcctttg | ccatgcaaacctgtcccaaca |
| Di 7NDL4 RT | tgatcgtttaatttttgttttgattgg | caccagaaataaccccaaaacacat |
| Di8 ND1 RT | gggggtattcgttcttgtgctc | aggagcccgatgcaaatcaa |
| Di 9ATP6 RT | tttttgcatcaggggcttcagtc | actgatacgcaaagtcaaagcaacacc |
| Di 10Cox2 RT | tgattgaattggttttgcaggtgttga | gccccacaaatctcagaacactgacc |
| Di 11ND3 RT | tgagtgtgggtttgatgtttgt | caaatcaccctgaaccaaaaa |
| Di 12ND5 RT | tggttgcctaaagctatggctgctc | tgcaactgaaccaaaacaggacaaca |
| Of-Ov 1ND2 RT | aatgttcctttgagtattactttt | caatacaaaacaaaacataaaca |
| Of-Ov 2ND4 RT | tgtaagtatgttgttttgtttg | tcataaaaaccaaaggtaaa |
| Of-Ov 3Cox1 RT | tctgttcttactatgcatggtgttt | tttgatccaaagtcacagcag |
| Of-Ov 5CytB RT | ctgctagttttactttaagttatatg | actagaactagaaccagtaaaatg |
| Of-Ov8 ND1 RT | gcgtgcttgtgttcagagtt | aaaccaataggcaaaaacaaaaa |
| Of-Ov 9ATP6 RT | ttttatatggagttgtgtaagttt | agctactcccctcatcaa |
| Of-Ov 10Cox2 RT | tcttttaagtttaatttgaagcgtag | ttatcaacatcaaataaccgaaa |
| Of 4ND6 RT | tttggattgggatccttcaaa | aaatccagaaccattaagcctaaaa |
| Of 6Cox3 RT | tgatggtcggggctggtatt | cattcactccaaaaatcgccaat |
| Of 7NDL4 RT | tcgtttgatttttgttttgttgg | tcaacaccataacccttaacacaga |
| Of 11ND3 RT | tttttggttccttttggtat | acaaaaccaaatcaacttacc |
| Of 12ND3 RT | tttttgggggttagtagttttt | aaaaacaaaaaccaatttgaga |
| Ov 4ND6 RT | tggattgggatccgttgaaaa | acaaactacgcatataaccgaaacca |
| Ov 6Cox3 RT | tcctttgatagttggggtgggta | catgcaaaccatgcaaaccag |
| Ov 7NDL4 RT | tttgttgggtattgaatttttgtttt | ccttatctacaccaaaacccttaaca |
| Ov 11ND3 RT | tgtgtcttttaaggatttttatggtgct | tcaacttaccaaaacaccactcca |
| Ov 12ND5 RT | gtgctcggaggggtgctatg | tgaaccaaaaccggagcaca |

“Filarial Mito” were used amplify portions of the mitochondrial genomes for sequencing. “Filarial Mito” primer sets work with various species, while “Av Mito,” “Cq Mito,” “Ll Mito,” and “Wb Mito” primer sets are specific to *A. viteae*, *L. loa* and *W. bancrofti*, respectively. “RT” primer sets were used to assess expression of protein coding genes.
